# Vision for Robust Robot Manipulation

**DOI:** 10.3390/s19071648

**Published:** 2019-04-06

**Authors:** Ester Martinez-Martin, Angel P. del Pobil

**Affiliations:** 1 RoViT, University of Alicante, 03690 San Vicente del Raspeig (Alicante), Spain; 2 RobInLab, Jaume I University, 12071 Castello de la Plana, Spain; 3 Interaction Science Dept., Sungkyunkwan University, Jongno-Gu, Seoul 110-745, Korea

**Keywords:** robotics, robot manipulation, depth vision

## Abstract

Advances in Robotics are leading to a new generation of assistant robots working in ordinary, domestic settings. This evolution raises new challenges in the tasks to be accomplished by the robots. This is the case for object manipulation where the detect-approach-grasp loop requires a robust recovery stage, especially when the held object slides. Several proprioceptive sensors have been developed in the last decades, such as tactile sensors or contact switches, that can be used for that purpose; nevertheless, their implementation may considerably restrict the gripper’s flexibility and functionality, increasing their cost and complexity. Alternatively, vision can be used since it is an undoubtedly rich source of information, and in particular, depth vision sensors. We present an approach based on depth cameras to robustly evaluate the manipulation success, continuously reporting about any object loss and, consequently, allowing it to robustly recover from this situation. For that, a Lab-colour segmentation allows the robot to identify potential robot manipulators in the image. Then, the depth information is used to detect any edge resulting from two-object contact. The combination of those techniques allows the robot to accurately detect the presence or absence of contact points between the robot manipulator and a held object. An experimental evaluation in realistic indoor environments supports our approach.

## 1. Introduction

Advances in Robotics are leading to a new generation of assistant robots working in ordinary domestic settings, such as healthcare and rehabilitation [1,2], agriculture [3], emergency situations [4,5], or guidance assistance [6]. In this context, the ability to autonomously manipulate objects is of critical importance. Though there exist a wide research on robot grasping (e.g., Refs. [7,8,9,10,11]), it is mainly focused on object location, along with motion and grasp planning. Only a few efforts have been devoted to monitoring the grasp action for error recovery, an issue that is, however, crucial to achieve the required level of autonomy in the robotic system.

Along this line, a state-of-the-art solution is to equip the robot gripper with tactile sensors. In this way, the presence or absence of a grasped object can be easily perceived through pressure distribution measure or contact detection [12,13]. For that reason, a wide variety of tactile sensors for robot hands have been developed [14]. However, the existing tactile technologies have multiple limitations. First, most of the existing sensors are too bulky to be used without sacrificing the system dexterity. Another reason is that they are too expensive, slow, fragile, sensitive to temperature, or complex to manufacture. They may also lack elasticity, mechanical flexibility or robustness. Therefore, it is necessary to have an alternative or complementary sensing approach to robustly detect errors in object grasping.

Alternatively, information about joint position, joint velocity or joint torque (*proprioception*), has been often used for robot grasping [15,16]. Nevertheless, the grasp stability may be affected by several parameters such as the configuration of the robotic gripper, the (mis)alignment of the joint axes, or inaccurate data (e.g., open/close instead of the exact grip aperture). These drawbacks limit the suitability of this approach for service robots.

As a solution, we propose to use computer vision since it can provide more accurate information than other robot sensors. Thus, the evaluation of a manipulation action may be mediated by a proper recognition of both the gripper and the held object. To the best of our knowledge, no other approach exists in which vision is used for error detection after an attempt to pick up an object. For instance, taking the Amazon Picking Challenge as a test case, none of the over 60 teams that participated in its three editions (2015–2017) reported the use of vision for detecting grasping errors [17,18]. Often grasping errors were not detected at all or error detection was based on a vacuum sensor when a suction cup was used [19], as well as weight checking [20].

A wide range of approaches for gripper and/or object recognition varying in complexity and functionality can be found in the literature. Currently, the most popular approach is *deep learning* [21,22,23,24,25,26]. This approach could be described as computational models composed of multiple processing layers that allows it to learn representations of data with multiple levels of abstraction. Nevertheless, as a training stage is required, all the manipulated objects (including the robot gripper) must be known in advance. In addition, the use of elastically deformable objects or grippers can lead to a failure of this approach since a sufficiently large number of visual appearances may not be available for system training. Furthermore, the high requirements of current deep learning solutions in terms of memory and computational resources make it infeasible for robot tasks.

With the purpose of real-time operation, visual local features could be used. One of the most implemented technique is SIFT [27,28]. This approach shares many features with neuron responses in primate vision. Basically, SIFT transforms visual input into linear scale-invariant coordinates that are relative to local features. In this way, an object can be located in an image that contains many other objects. The main drawback of this approach (and its alternatives [29,30,31]) is that a certain amount of texture in the objects to be detected is required, a requirement that cannot be always guaranteed in ordinary, domestic settings. Moreover, the grasping action may result in a great object occlusion making the object visually undetectable.

In this context, traditional Computer Vision techniques could fit since they allow us to extract simple image features like colour or shape that can be used for a proper robot gripper monitoring. In particular, similarly to the human vision system, this paper proposes a technique to combine simple visual features (e.g., motion, orientation, colour, etc.) for gripper monitoring. More specifically, edge, depth and colour are properly combined to detect a contact between a robot gripper and any grasped object.

This paper is organised as follows: Section 2 overviews the robot grasp task, while Section 3 introduces our approach for grasping monitoring. Experimental results are presented and discussed in Section 4. Finally, conclusions and future work can be found in Section 5.

## 2. The Grasping Task

**Figure 1:**
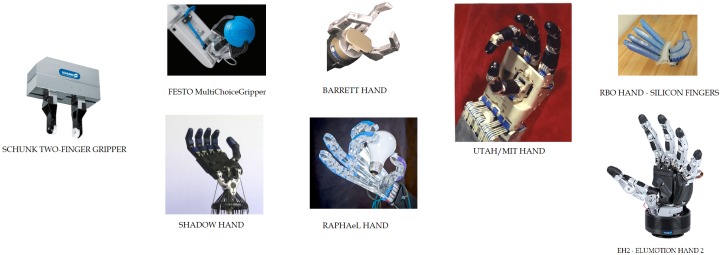
A sample of the evolution of robotic manipulators.

**Figure 2:**
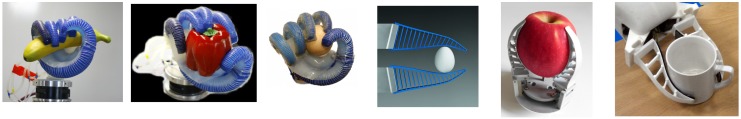
A sample of deformations when flexible robotic grippers grasp an object.

Any grasping task involves a device to hold and manipulate objects that can be in the form of simple grippers or highly dexterous robotic hands (see Figure 1 for some examples). So, these devices have evolved according to the Robotics demands. Firstly, the two-finger grippers were designed to satisfy the industrial assembly needs. From that starting point, different designs have been proposed in the literature to properly fulfill robot service tasks. In addition, the wide variety of objects to deal with has also led to the use of different materials allowing the robot manipulator to flexibly adapt itself to the most varied shapes (see Figure 2). This flexibility results in a deformation (sometimes permanent) and, as a consequence, recognizing the gripper turns into a much more difficult task. In addition, techniques based on a model of the gripper or its shape become impractical due to the complexity in modelling the many different ways a gripper or its fingers can deform.

**Figure 3:**
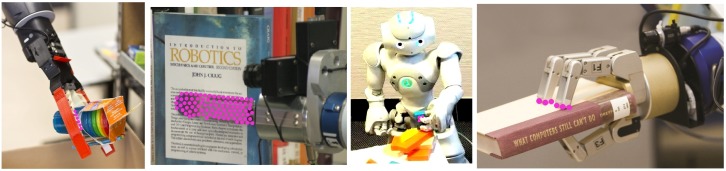
Contact points resulting from robotic grippers grasping an object.

**Figure 4:**
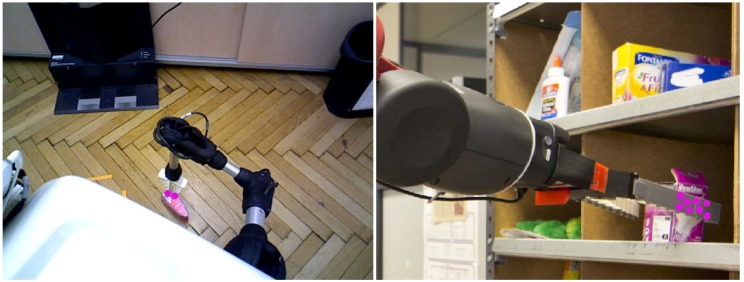
*Tricky* RGB situations of non-grasping contact points where a visual aligment between an object and the robotic manipulator can be confusing.

For that reason, an abstraction is required. Generally speaking, a *grasp* can be defined as a set of contacts between a robot manipulator and the surface of any held object (see Figure 3). From this definition, the grasping action could be detected as the contact between the object and the manipulator. Therefore, a solution could be to properly detect both the object and the manipulator and find their contact points. However, there are several issues to be overcome such as detecting them in different environments, the wide variety of objects (some of them could be quite similar to the others), and a great manipulator diversity. In addition, using only an RGB input can lead to *tricky* situations where the manipulator and the object are not in contact, but the visual system may wrongly identify contact points. As illustrated in Figure 4, given the visual alignment between the robotic manipulator and the object, the robot may be unable to distinguish if they are in contact or not. What is more, colour-based object recognition highly depends on illumination conditions; so, with the purpose of reducing its influence, different colour models have been investigated in terms of sensitivity to image parameters [32]. From this study, the *Lab* colour space is the best alternative due to its invariance under different conditions.

**Figure 5:**
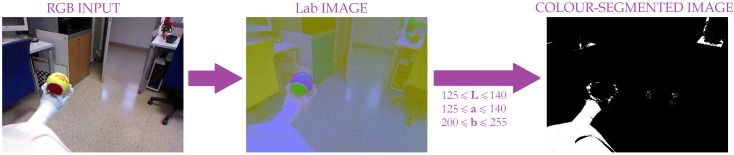
Image segmentation based on *Lab* colour model.

Note that the colour coordinates are experimentally set for each robotic manipulator. For that, several images under different environmental conditions (five images in our experiments) are required to properly adjust the *Lab* range. However, a colour-based segmentation extracts all the elements within the scene satisfying those colour coordinates, as illustrated in Figure 5. Thus, more information is required to properly identify the robot gripper so that a robust detection of grasp contact points is achieved and, as a consequence, the grasping action itself is more dependable. In this paper, we propose to fuse *Lab* data with depth information to achieve this goal. This data could be obtained from an RGB-D camera, a popular device in the last years due to its low price and the enriched information it provides. As explained in the following section, this sensory fusion also solves the detection of the gripper and the object.

## 3. Grasp Monitoring

As mentioned above, the proposed approach is based on the fusion of two visual inputs: *RGB* and depth. So, *RGB* information provides an early, coarse image segmentation. As previously shown in Figure 5, the *RGB* input is first converted into its corresponding *Lab* image. Then, a segmentation based on *Lab* gripper coordinates is applied. Given that real environments are considered, several elements could present the same colour distribution and, as a consequence, they also appear in the segmentation result. This is the case of Pepper’s robot that is homogeneously coloured and consequently, all the robot parts are present in the colour-based segmentation result as depicted in Figure 5. For that reason, an additional cue is required to properly identify the robot gripper and, therefore, the grasping task.

**Figure 6:**
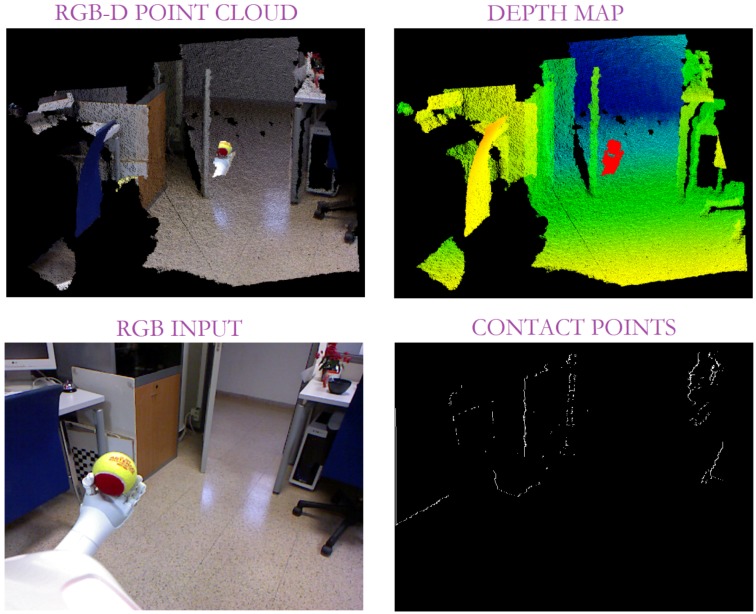
Contact points detected by the depth-based map.

In this sense, depth data has been used to overcome the colour segmentation issues. Thus, on the one hand, the depth cue provides information about an object’s position with respect to its neighbours. This allows the robot to robustly detect the contact points (or their absence) between the scene objects. In this way, the real contact points can be properly identified based on the depth difference between two touching objects. Nonetheless, this approach detects any contact point between two objects. So, for instance, apart from the grasping contact points, it obtains the contact points between a table and any object on it, those between two objects in touch or overlapping, or even the contact points between different parts of the same object, as shown in Figure 6. Due to the sensor limitation, there is a noteworthy amount of pixels without depth information. For example, too close pixels like the robot’s body, are missing in the depth map. In addition, other visual objects are *vanished* as it is the case of the door. As a result, the number of contact points is reduced although more information is necessary to accurately isolate gripper-object contact points.

**Figure 7:**
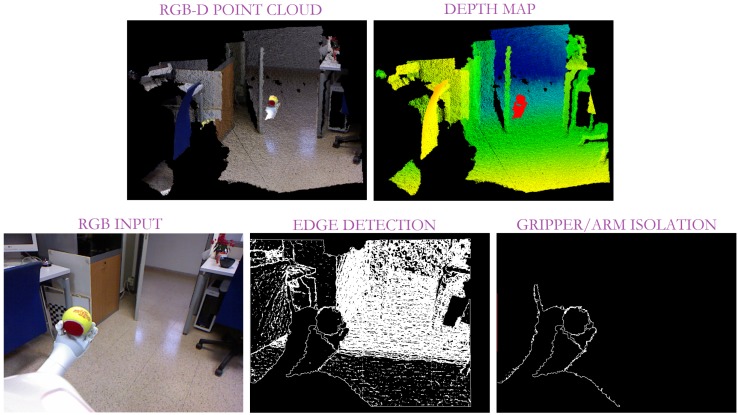
Results of our edge detection from depth information such that the bottom centre column represents the first contour segmentation, while the last one shows the contour segmentation after refinement.

To overcome this difficulty, the gripper recognition is applied to properly identify the grasping contact points and, consequently, evaluate the robot grasping task and detect its possible errors. With that aim, a contour extraction is performed, that is, the contours are obtained from depth changes. A pixel is classified as a contour when there is a leap between the depth information for that pixel and one of its neighbours. In our case, that jump was limited to 0.01 depth units (approximately 1 cm). Note that to achieve this, a critical issue is the missing depth points mainly resulting from the distance with respect to the sensor and the object’s thinness. As a solution, the border pixels in terms of presence/absence of information have been also considered as contours (see Figure 7).

This fact leads to all the object’s contours in the scene. Consequently, an edge refinement is necessary to adequately isolate the robot gripper. Given that the vision system is always located at the top of the robot and looking ahead, the robot actuator contour emerges from the bottom part of the image. Therefore, all the contours out of the image bottom are discarded as shown in Figure 7.

Once the contours are obtained, they are combined with the colour segmented image. In this way, the gripper is properly identified within the visual scene. The last step is to check the presence or absence of contact points with a held object. For that, only the objects contained between the robot *fingers* are considered.

**Figure 8:**
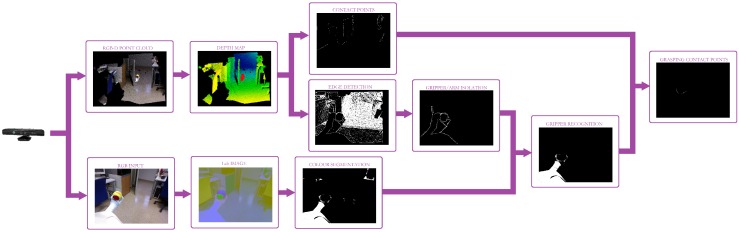
Our approach flowchart for robust grasping monitoring.

Therefore, the whole approach combines all the abovementioned methods to properly check the grasping status at any time. So, as illustrated in Figure 8 and sketched in Algorithm 1, our approach concurrently performs three raw segmentations: the first is based on the Lab gripper components; the second obtains all the contact points between two objects separated by less than 5 cm, while the last one outputs an image with all the object contours. As all the object contours are obtained, the last segmentation is refined such that only the ones that start at the bottom of the image are considered. This information, together with the colour segmentation, allows the system to properly isolate the robot gripper. Finally, the overlap between this last image and the raw contact points segmentation provides the robot with the information about the presence or absence of contact points and, consequently, the status of the grasping task. Note that the proposed approach only depends on two parameters: the *Lab* components, corresponding to the robot gripper; and the depth threshold. So, on the one hand, the *Lab* components are defined by an interval of values for each component obtained from a Lab-component analysis of the robot gripper under different illumination conditions. On the other hand, the depth threshold must be set from camera information such that it approximately corresponds to 1 cm.

**Algorithm 1:** Our grasping monitoring approach.
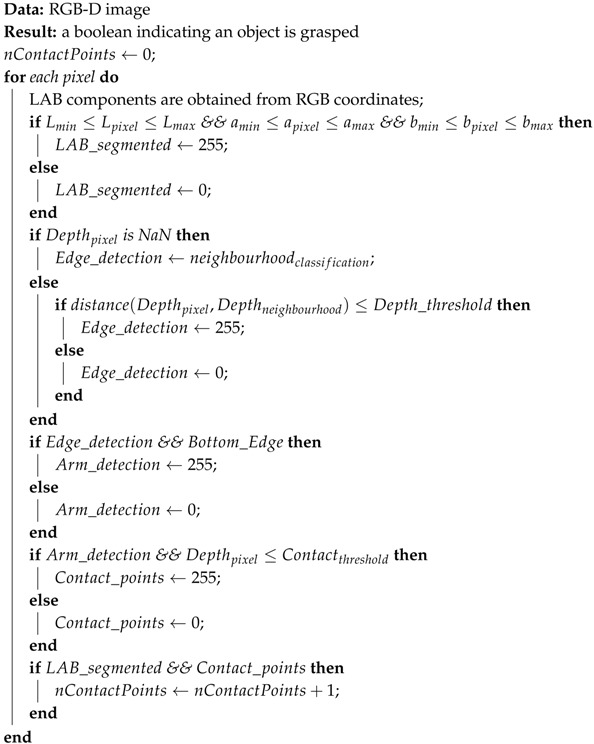


## 4. Experimental Results

**Figure 9:**
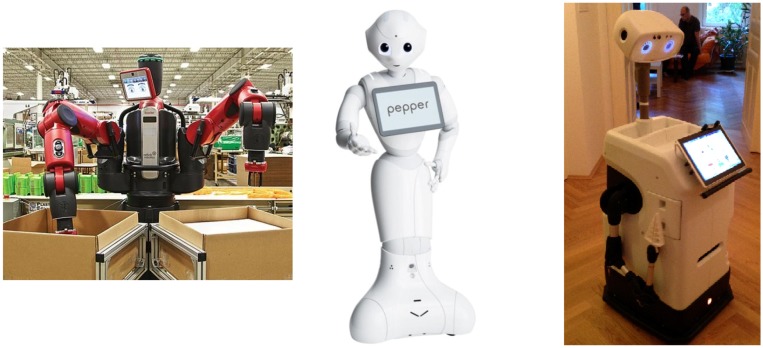
The three robot platforms used to evaluate the performance of our approach: the *Baxter* robot [33] (left), the *Pepper* robot [34] (center) and the *Hobbit* robot [35] (right).

With the purpose of validating our approach, three different robot platforms have been used: the *Baxter* robot [33], the *Pepper* robot [34] and the *Hobbit* robot [35] (see Figure 9). The *Baxter* robot is a two-armed robot designed for industrial automation. On the contrary, the *Pepper* and *Hobbit* robots are social platforms designed to interact with people. So, *Pepper* is a commercial semi-humanoid robot being adapted to several applications like a guide assistant, while the *Hobbit* is a socially assistive robot aimed at helping seniors and elderly people at home. All these robot platforms are endowed with multiple sensors, providing the robot with perceptual data, and actuators, allowing the system to perform its tasks.

There are several differences between them to be taken into account for grasping tasks. On the one hand, the robot gripper is quite different in each robot. In particular, *Baxter* is provided with a parallel jaw gripper intended to perform industrial tasks such as packaging, material handling or machine tending. On its behalf, *Pepper* emulates a human hand with a five-finger gripper, whereas *Hobbit* is endowed with a gripper based on FESTO *Fin Ray Effect*; in this design, the two soft, triangular fingers with hard crossbeams can buckle and deform to conform around grasped objects. This allows us to evaluate the performance of our approach not only with rigid grippers but also with continuously shape-changing grippers.

On the other hand, the camera location varies between the platforms. Indeed, the visual input is provided by a pan-tilt RGB-D camera (i.e., Microsoft Kinect) mounted on the *head* of each robot and, therefore, it is approximately located at a height of 160 cm (*Baxter*), 110 cm (*Pepper*), and 130 cm (*Hobbit*).

**Figure 10:**
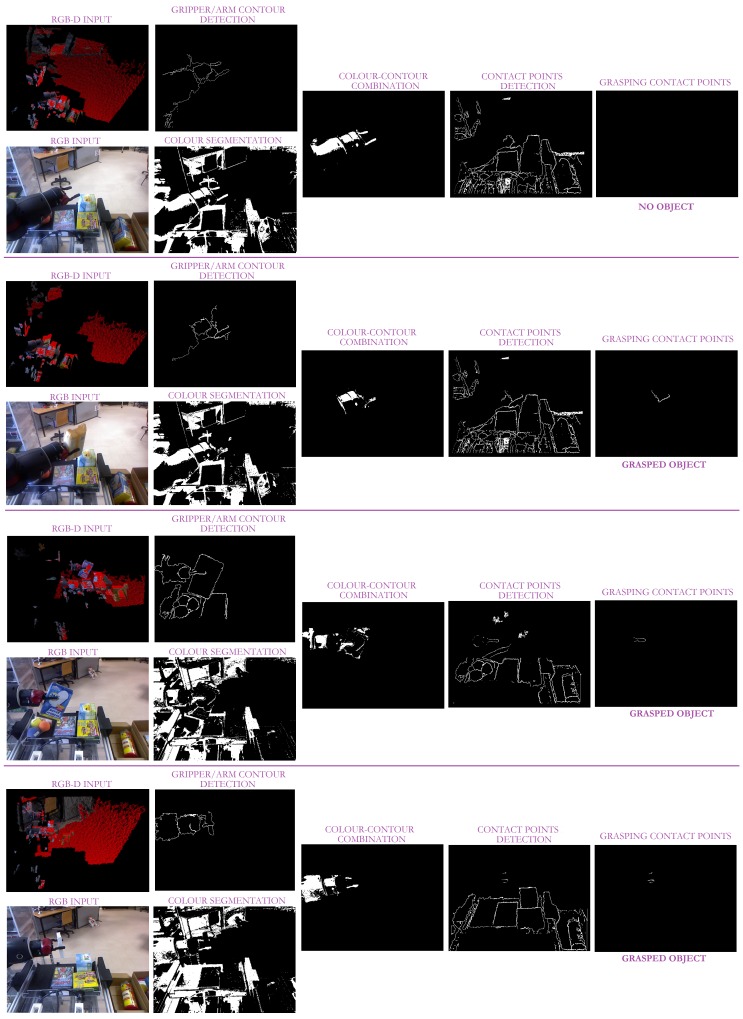
Some experimental results of our approach with the *Baxter* robot in pick-and-place tasks. The first column corresponds to the taken RGB-D image given as an RGB image and a depth map. The second column illustrates the Lab segmentation with the robot arm contour obtained from the contour segmentation refinement. The third column illustrates the combination of the images in the two columns. The next column depicts all the two-object contact points based on depth proximity. The last image shows the contact points obtained from the overlap between the results in the third and fourth columns.

**Figure 11:**
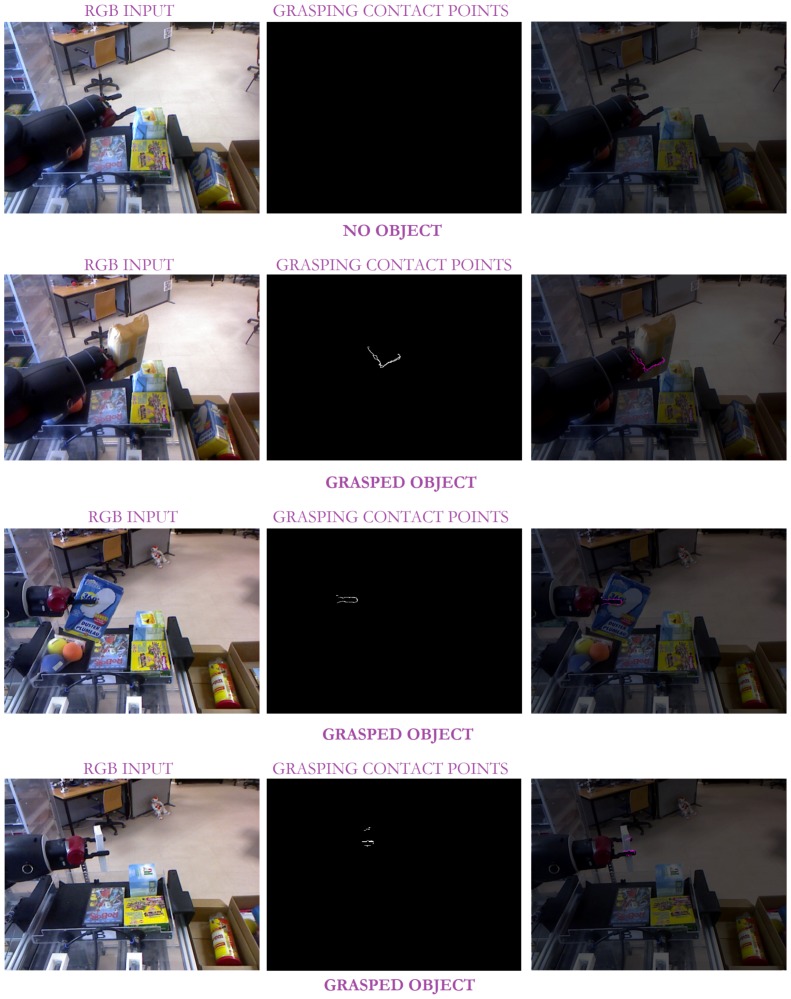
Some experimental results of our approach with the *Baxter* robot in pick-and-place tasks: the left column corresponds to the input RGB image; the middle column illustrates the detected contact points; and the last column shows the overlapping between the original image and the detected contact points (in pink).

**Figure 12:**
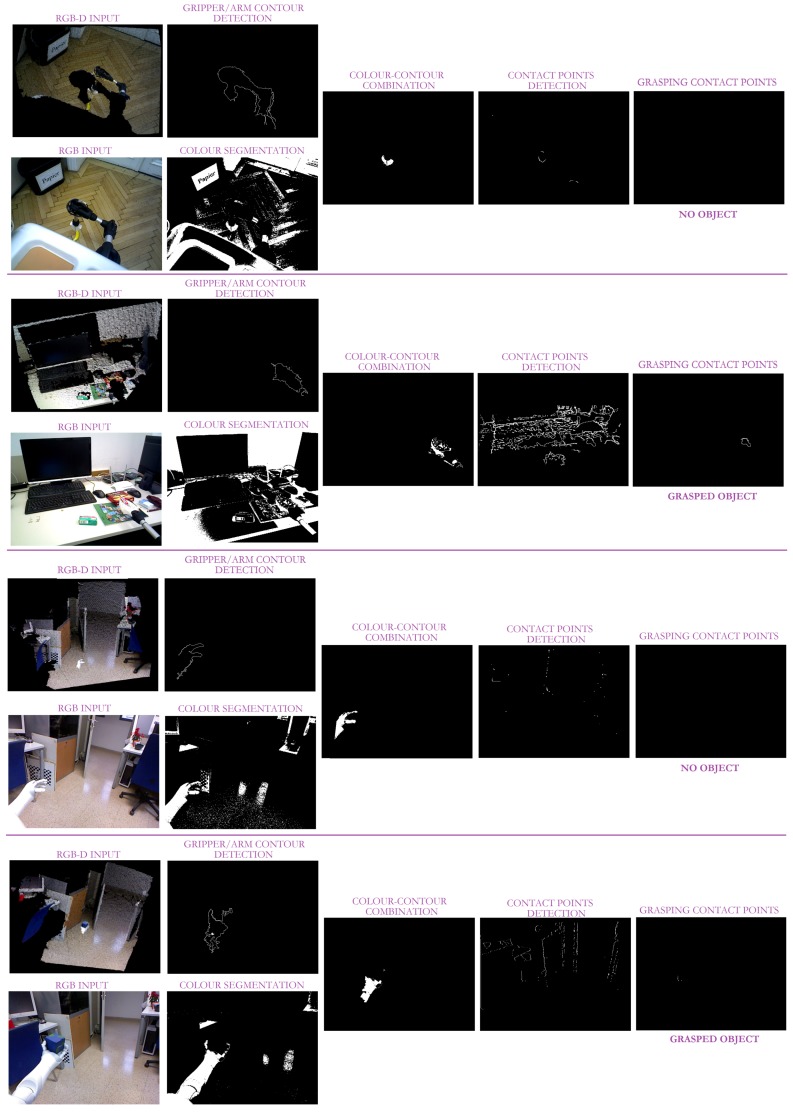
Some experimental results of our approach with the *Hobbit* and *Pepper* robots in assistive tasks. The first column corresponds to the taken RGB-D image given as an RGB image and a depth map. The second column illustrates the Lab segmentation with the robot arm contour obtained from the contour segmentation refinement. The third column illustrates the combination of the images in the two columns. The next column depicts all the two-object contact points based on depth proximity. The last image shows the contact points obtained from the overlap between the results in the third and fourth columns.

**Figure 13:**
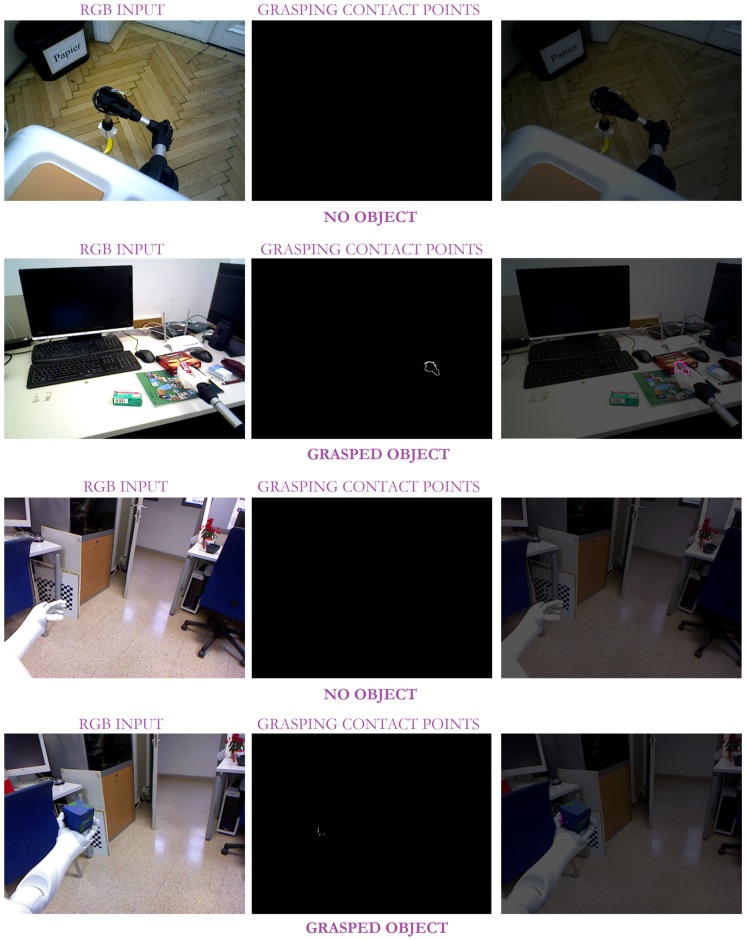
Some experimental results of our approach with the *Hobbit* and *Pepper* robots in assistive tasks: the left column corresponds to the input RGB image; the middle column illustrates the detected contact points; and the last column shows the overlapping between the original image and the detected contact points (in pink).

With the aim to accurately evaluate the approach performance, the three robots were located at different unstructured scenarios (seven in total) carrying out different tasks. So, *Baxter* is performing a pick-and-place task (see Figure 10 and Figure 11), while *Pepper* and *Hobbit* execute assistive tasks as depicted in Figure 12 and Figure 13. A total of twenty objects were used in our experiments including challenging ones such as keys, a bottle of water, a pack of gum, or a headphone’s bag.

As shown in Figure 10, several contact points are detected within a scene. So, all the objects on the bin present contact points. However, thanks to the gripper recognition module, only the *grasping* contact points are considered for evaluating the status of the grasping task. Another critical issue is the missing depth data, clearly present in Figure 10. The combination of colour and depth cues and the inclusion of non-data points allows our approach to successfully detect the presence or absence of contact points between the robotic manipulator and the object, as shown in Figure 10 and its partial version in Figure 11.

On its behalf, Figure 12, and the partial version in Figure 13, highlight the resolution of the visual ambiguities since no false positive *grasping* contact points are obtained, even when the robot gripper is close to the ground and its visibility is poor. Thin objects can be also properly detected when they are grasped as in the case of the chewing gum pack. In addition, it can be observed that neither the changing shape of *Hobbit*’s gripper nor the use of different robot grippers affect the approach results.

The approach’s performance has been analysed by means of a comparison between its output in terms of presence or absence of a grasped object and the images manually labelled considering seven scenarios, three robot platforms, and twenty objects with different visual features. With a total of one thousand 640 × 480 images, the algorithm was able to successfully evaluate the grasping status with an accuracy of 97.5% at a speed of 160 ms per image. Note that this speed allows the robot to work in real-time, what is crucial for service robots. The main errors were a consequence of handling small and/or thin objects in specific configurations.

## 5. Conclusions

Reliable grasping is a decisive task for any robotic application from industrial pick-and-place to service assistance. For that reason, it is critical to successfully perform any grasp and properly recover for any error. This is, however, not straightforward due to the great variety of robot manipulators and, especially, those with a design that prevents the use of other devices like touch sensors.

In this paper, we propose a novel vision approach for monitoring the grasping tasks and verifying any lost of the held object. The underlying idea is the recognition of the contact points between the robot manipulator and the grasped object. For that, all the contact points between two objects within the scene are obtained from depth data. Then, it is checked whether any contact point corresponds to the inner part of the gripper. With that aim, a griper recognition method based on the fusion of depth and colour cues is presented.

So, on the one hand, the input RGB image is segmented according to the *Lab*-colour manipulator coordinates. At the same time, edge information is extracted from depth data. An edge refinement under the assumption of the manipulator boundary comes from the bottom of the image, allows our approach to extract the robot arm contour. Finally, the colour-contour combination together with the contact point map determines the grasping status at any time.

With the aim of properly evaluating the performance of our approach, three different robot platforms have been used: Baxter, Pepper and Hobbit. So, its performance was evaluated in different scenarios, with different objects and with several head poses. The experiment results highlight the good performance, obtaining an accuracy of 97.5%. It is noteworthy that the erroneous cases are present when thin or small objects are manipulated and only in some manipulator configurations. For that reason, the approach should improve to cover these cases. In addition, the proposed approach runs in real-time, which is an issue particularly problematic for robot applications.

As future work, other visual features will be analysed with the aim of overcoming the problems detected with small or thin objects without constraining the robot’s autonomy.
